# Exploring the Potential of Exosome-Related LncRNA Pairs as Predictors for Immune Microenvironment, Survival Outcome, and Microbiotain Landscape in Esophageal Squamous Cell Carcinoma

**DOI:** 10.3389/fimmu.2022.918154

**Published:** 2022-07-08

**Authors:** Fangchao Zhao, Zhirong Li, Zefang Dong, Zengying Wang, Pengfei Guo, Dengfeng Zhang, Shujun Li

**Affiliations:** ^1^Department of Thoracic Surgery, The Second Hospital of Hebei Medical University, Shijiazhuang, China; ^2^School of Clinical Medicine of Hebei Medical University, Shijiazhuang, China; ^3^Clinical Laboratory Center, The Second Hospital of Hebei Medical University, Shijiazhuang, China; ^4^Department of Ophthalmology, The Third Hospital of Hebei Medical University, Shijiazhuang, China

**Keywords:** esophageal squamous cell carcinoma, exosome, long non-coding RNA, tumor immune microenvironment, prognostic model

## Abstract

Accumulating studies have demonstrated the indispensable roles of exosomes and long non-coding RNAs (lncRNAs) in cancer progression and the tumor microenvironment (TME). However, the clinical relevance of exosome-related lncRNAs (ER-lncRNAs) in esophageal squamous cell carcinoma (ESCC) remains unclear. Three subtypes were identified by consensus clustering of 3459 valid ER-lncRNA pairs, of which subtype A is preferentially related to favorable prognosis, lower stromal and immune scores, and higher tumor purity scores. Higher immune cell infiltration, higher mRNA levels of immune checkpoints, higher stromal and immune scores, and lower tumor purity were found in subtype C, which presented a poor prognosis. We developed a prognostic risk score model based on 8 ER-lncRNA pairs in the GEO cohort using univariate Cox regression analysis and LASSO Cox regression analysis. Patients were divided into a high risk-score group and low risk-score group by the cut-off values of the 1-year ROC curves in the training set (GEO cohort) and the validation set (TCGA cohort). Receiver operating characteristic (ROC) curves, Decision curve analysis (DCA), clinical correlation analysis, and univariate and multivariate Cox regression all confirmed that the prognostic model has good predictive power and that the risk score can be used as an independent prognostic factor in different cohorts. By further analyzing the TME based on the risk model, higher immune cell infiltration and more active TME were found in the high-risk group, which presented a poor prognosis. Patients with high risk scores also exhibited higher mRNA levels of immune checkpoints and lower IC50 values, indicating that these patients may be more prone to profit from chemotherapy and immunotherapy. The top five most abundant microbial phyla in ESCC was also identified. The best ER-lncRNAs (AC082651.3, AP000487.1, PLA2G4E-AS1, C8orf49 and AL356056.2) were identified based on machine learning algorithms. Subsequently, the expression levels of the above ER-lncRNAs were analyzed by combining the GTEx and TCGA databases. In addition, qRT-PCR analysis based on clinical samples from our hospital showed a high degree of consistency. This study fills the gap of ER-lncRNA model in predicting the prognosis of patients with ESCC and the risk score-based risk stratification could facilitate the determination of therapeutic option to improve prognoses.

## Introduction

In 2020, there were 604,000 new cases of esophageal cancer and 544,000 deaths worldwide, ranking 7th and 6th in incidence and mortality of malignant tumors, respectively (1). The number of esophageal cancer cases and deaths in China and their global percentages were 324,000 (53.7%) and 301,000 (55.3%), respectively (2). Esophageal cancer is more malignant, prone to recurrence and metastasis, and has a poor prognosis (3). There are two main histological types of esophageal cancer: esophageal squamous cell carcinoma (ESCC) and esophageal adenocarcinoma, of which ESCC is the common histological type of esophageal cancer in China, accounting for more than 80% (4). Despite the increasing maturity of esophageal cancer treatment technology, the overall 5-year survival rate is still below 20% (5). Therefore, there is an urgent need for research and development of new prognostic biomarkers for ESCC.

Exosomes are extracellular vesicles of 40-100 nm in diameter with a lipid bilayer membrane structure, carrying the contents of proteins, lipids and nucleic acids, which are involved in various physiological processes such as immune response, antigen presentation, intercellular communication and protein and RNA transport (6, 7). At the same time, exosomes are widely distributed in various body fluids, including blood, urine, amniotic fluid, and malignant ascites, enabling the cells to perform biological functions at distant locations (8, 9). Recent studies have shown that exosomes play a crucial role in tumorigenesis, development and metastasis (10). For example, exosomes from breast and prostate cancer cells induce tumor formation by transferring their miRNAs (11, 12). Exosomes are also associated with angiogenesis and extracellular matrix remodeling in the tumor microenvironment, which are key steps in tumor growth and metastatic spread. For example, exosomes from hypoxic glioblastoma (GBM) cells induce pro-angiogenic processes in endothelial cells and proliferation of GBM cells (13). In addition, exosomes shed by cancer cells promote resistance to various chemotherapeutic agents and antibodies. For example, binding of cancer-associated fibroblast (CAF)-derived exosome miR-21 to APAF1 in ovarian cancer cells confers paclitaxel resistance to cancer cells (14).

Long non-coding RNAs (lncRNAs) is an RNA molecule consisting of more than 200 nucleotides and has no protein-coding ability. LncRNAs are aberrantly expressed in various cancers and are involved in tumorigenesis and progression (15). Recent studies have shown that lncRNAs can be carried by exosomes, are widely involved in intercellular material exchange and signal transduction and are stably present in body fluids, and are not affected by their degradation by endogenous RNA enzymes (16). In addition, exosome-derived lncRNAs can regulate tumor cell apoptosis (17), proliferation and migration and induce angiogenesis (18). The dysregulation of exosomal lncRNAs could affect the tumor microenvironment (TME) and regulate critical oncological behaviors (19). These exosome-derived lncRNAs have potential as diagnostic and prognostic biomarkers of various cancers (20). However, there is still a lack of bioinformatic research on exosome-related lncRNAs (ER-lncRNAs) in ESCC.

In the present study, we systematically explored the prognostic significance and the TME heterogenicity of ER-lncRNAs in ESCC. This study may provide new guidance on survival outcomes and treatment strategies for ESCC.

## Materials and Methods

### Processing of Transcriptome Data

The RNA sequencing data and clinical information of ESCC patients were obtained from the TCGA database (80 tumor and 11 normal samples) and the GEO database (GSE53624, 119 tumor samples). The number of transcriptional samples (Stage I-II: 53 samples; Stage III-IV 64samples) is balanced in the GEO dataset. In each dataset, duplicate sequencing samples from the same patient were excluded, and patients lacking complete follow-up information and with zero survival days were excluded. For TCGA-ESCC cohort, convert RNA-seq data in FPKM format to TPM. The GEO cohort is annotated by the Affymetrix platform. The ComBat algorithm in the “sva” package is used for batch effect elimination (meta queue) for the TCGA and GEO databases. The GENCODE database (GRCh38 version) is used for lncRNA and mRNA annotation, taking the intersection of the two platforms. 121 exosome-related genes (ER-mRNAs) were downloaded from the ExoBCD database (https://exobcd.liumwei.org/), and finally 94 ER-mRNAs could be annotated in the Meta cohort. The above ER-mRNA was used to screen exosome-related lncRNAs (ER-lncRNAs) by co-expression strategy with correlation coefficient > 0.4 and p-value < 0.001 as the screening threshold. The “limma” package was used for differential expression analysis between ER-lncRNAs and ER-mRNAs with thresholds set to logFC >1 and FDR <0.05 Real.

### ER-lncRNA Pairs

**Figure 1:**
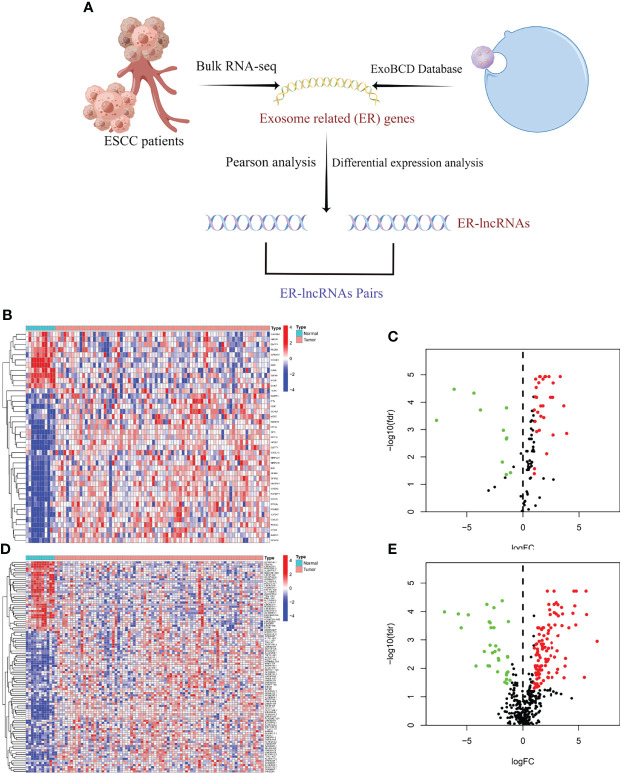
Selection of ER-lncRNAs. **(A)** The flow chart of ER-lncRNA selection. **(B, C)** Heatmap and Volcano plot of differentially expressed ER-mRNAs between tumor tissues and normal tissues. **(D, E)** Heatmap and Volcano plot of differentially expressed ER-lncRNAs between tumor tissues and normal tissues.

Cyclic pairing was performed on the differential ER-lncRNAs, assuming C=lncRNA-A + lncRNA-B. If the expression level of lncRNA-A is higher than that of lncRNA-B, the result of C is 1, otherwise the result of C is 0, and finally a new matrix (0 or 1) containing C is generated. If the expression level of a certain lncRNA in ER-lncRNA Pairs is 0 or 1, the relationship between Pairs and prognosis is not considered. A valid match was considered when the number of ER-lncRNA Pairs expressed as 0 or 1 accounted for more than 20% of the total number of pairs. The flow chart is shown in **Figure 1A**.

### Consensus Clustering

According to the expression profiles of ER-lncRNA, consensus clustering was performed using the “ConsensusClusterPlus” package in R by setting the number of groups to 9, 100 replicates, and the pltem=0.8. The optimal cluster number was calculated using the consensus matrix and cumulative distribution function (CDF). The differences in the overall survival (OS) between different clusters were estimated using the Kaplan-Meier method. Comparisons of the distribution of categorical data among the clusters were done using the chi-squared test.

### Enrichment Analysis

The GSVA method was used to assess differences in biological pathways between subtypes. Differential pathways between different subtypes were analyzed using the “limma” package, with c2.cp.kegg.v7.0.symbols.gmt as the reference gene set and FDR < 0.05 as the screening threshold.

### Construction and Validation of Risk-Score Model

Univariate Cox regression analysis was performed for all ERlncRNA pairs (*P* < 0.001), followed by least absolute shrinkage and selection operator (LASSO) Cox regression analysis (10-fold cross-validation). LASSO regression was run for 1000 cycles, and for each cycle, 1000 random times were set to remove redundant ER-lncRNA pairs. We included the above ER-lncRNA pairs in the Cox regression risk model. When the AIC value was minimal, the calculation was stopped and the model was also used as the best model. The risk score was calculated as follows:


$$\sum_{\text{i}=1}^{k}{\text{βi}}_{\text{i}}*{\text{S}}_{\text{i}}$$

The cut-off values were determined by evaluating the 1-year ROC curves in the training set (GEO cohort) and the validation set (TCGA cohort). Patients were divided into high-risk and low-risk groups according to the cut-off values. Subsequently, Kaplan-Meier curves and ROC curves were applied to assess the prognostic role of the model. To verify the clinical application value of the constructed model, we analyzed the association between the model-based risk score and clinicopathological features based on the GEO database. Additionally, univariate and multifactor Cox regression analyses were performed to determine whether the model could be used as an independent predictor of clinical prognosis.

### Immune Infiltration Analysis

The ssGSEA algorithm was used to calculate the scores of 29 immune cells and immune function in ESCC samples, and the “pheatmap” package was used for heatmap visualization. The ESTIMATE algorithm identifies specific features associated with stromal cell and immune cell infiltration. Differences between different molecular subtypes and risk groups were compared using the Kruskal Wallis test. We used TIMER, XCELL, MCPcounter, EPIC algorithms to analyze the relationship between risk scores and immune cell characteristics. Additionally, we calculated enrichment scores for immune microenvironment-related pathways in each ESCC sample using the “IOBR” package.

### Drug Sensitivity Analysis

IC50 was calculated using the “prophetic” package in R software, and chemotherapeutic drugs were obtained from the Genome of Drug Sensitivity in Cancer (GDSC) database.

### Tissue Samples and Quantitative Real-Time Polymerase Chain Reaction

A total of 10 tumor tissue samples and nearby normal esophageal tissue samples were obtained from ESCC patients who underwent tumor resection. All tissue samples were collected from the Thoracic Surgery Department of the Second Hospital of Hebei Medical University with the approval by the Medical Ethics Committee of the hospital. The specific experimental protocol comes from our previous research (21, 22).

### Statistical Analysis

All the statistical analyses were made with the R software (v.4.0.1). The above section has described detailed statistical approaches for transcriptome data processing. A p-value less than 0.05 was of statistical significance.

## Results

### Identification of ER-lncRNAs

We identified 41 differentially expressed ER-mRNAs in normal and tumor samples in the meta cohort following the details described in the methods (**Figures 1B, C**). Subsequently, co-expression analysis was performed between differentially expressed ER-mRNAs and lncRNAs in tumor samples from the meta cohort, and a total of 620 ER-lncRNAs were identified. Ultimately, secondary differential analysis was performed in different samples, and 142 were defined as differentially expressed ER-lncRNAs (**Figure 1D**), of which 109 were upregulated and 33 were downregulated (**Figure 1E**).

### Identification of Molecular Subtypes Based on ER-LncRNA Pairs

**Figure 2:**
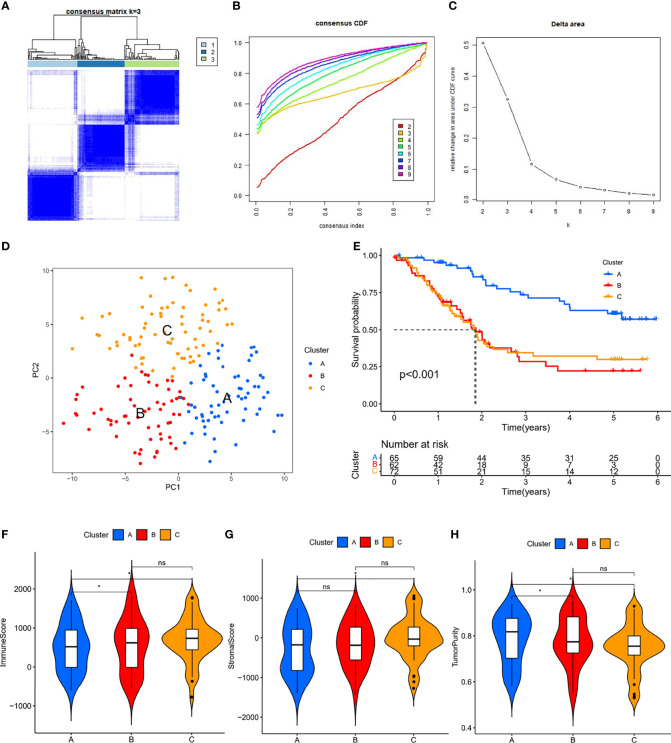
ER-lncRNA subtypes. **(A)** Consensus clustering matrix when k = 3. **(B)** Consensus clustering CDF with k valued 2 to 9. **(C)** Relative change in area under the CDF curve for k = 2 through 9. **(D)** PCA analysis showed that ESCC could be well differentiated into three subtypes based on the expression of ER-lncRNA. **(E)** Kaplan-Meier curves of OS for three subtypes in ESCC. Differences in immune score **(F)**, stromal score **(G)** and tumor purity **(H)** between different subtypes. ns, not significant, *P < 0.05.

According to the method described, we identified 3459 valid ER-lncRNA pairs in the meta cohort (**Supplementary File 1**). These ER-lncRNA pairs were further used for clustering analysis. The cluster effect was best when ESCC patients were clustered into three subtypes, and the subtype internal consistency and stability were good (**Figures 2A–C**). PCA analysis revealed significant heterogeneity in molecular typing based on ER-lncRNA pairs (**Figure 2D**). Survival analysis showed that subtype B had the worst prognosis of these three molecular subtypes, whereas subtype A had the best prognosis (**Figure 2E**). The ESTIMATE algorithm was used to assess stromal score, immune score and tumor purity for the three subtypes. As shown in **Figures 2F–H**, subtype C had the highest stromal and immune score and the lowest tumor purity score, while subtype A had the lowest stromal and immune score and the highest tumor purity score.

### Immunological Characterization of Molecular Subtypes and Drug Sensitivity Analysis

**Figure 3:**
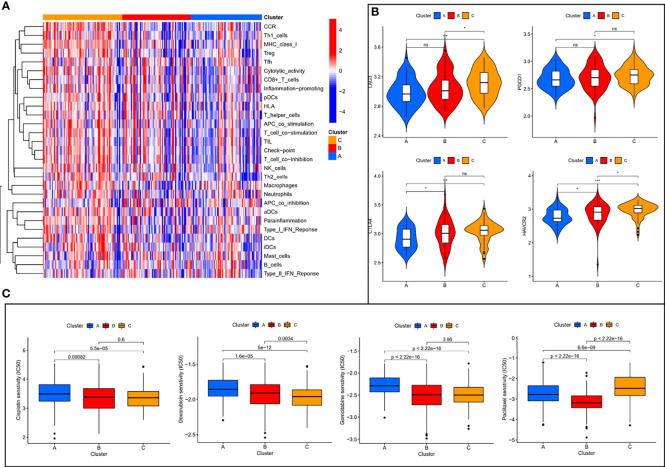
Immune characterization analysis and drug sensitivity analysis of molecular subtypes. **(A)** The expression of immune function between different subtypes. **(B)** The expression levels of immune checkpoint genes between different subtypes. **(C)** The boxplot of sensitivity of common chemotherapy drugs in different subtypes. ns, not significant, *P < 0.05, **P < 0.01, ***P < 0.001.

Of the three molecular subtypes, subtype C showed the highest enrichment and activity in several immune cells, pathways or functions (**Figure 3A**). Subsequently, we examined the expression of four immune checkpoint genes (i.e., PDCD1, CTLA4, HAVCR2, and LAG3) and found that the mRNA levels of most of the immune checkpoints were higher in subtype C than in other subtypes, which may suggest that this subtype may benefit more from immunotherapy (**Figure 3B**). In addition, we used the pRRophetic algorithm to evaluate the therapeutic effect of chemotherapy drugs. Among some classical chemotherapy drugs, subtype B was more sensitive to cisplatin, doxorubicin, gemcitabine, paclitaxel (**Figure 3C**), suggesting that molecular subtype-based grouping may play a guiding role in chemotherapy.

### Biological Features of Molecular Subtypes

**Figure 4:**
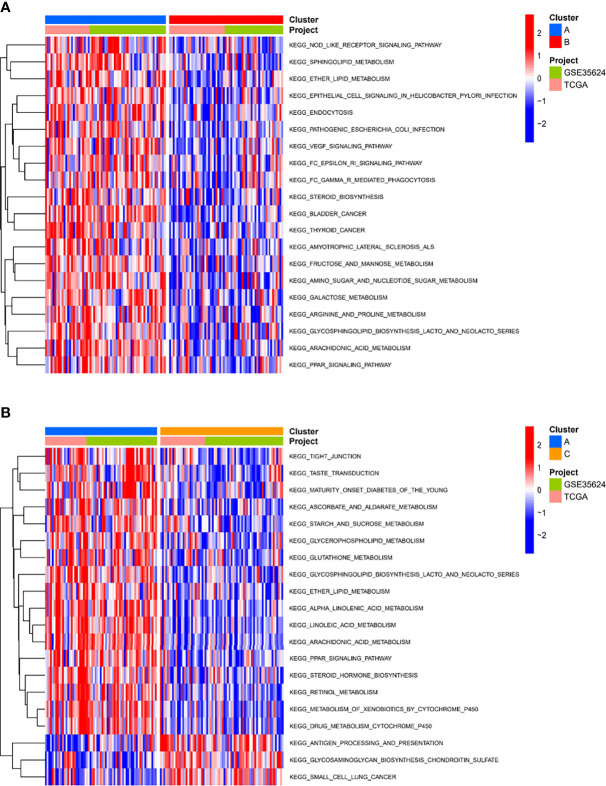
Biological characterization of molecular subtypes. **(A, B)** The GSVA pathway enrichment analysis between different subtypes.

To investigate the causes of the different survival states and immune landscapes, we used GSVA to study the biological processes between the different subtypes. The results showed that subtype A had more activated pathways compared with subtype B, such as KEGG_NOD_LIKE_RECEPTOR_SIGNALING_PATHWAY, KEGG_SPHINGOLIPID_METABOLISM, KEGG_ETHER_LIPID_METABOLISM, etc. (**Figure 4A**). Compared with subtype C, subtype A has more activated pathways, such as KEGG_TIGHT_JUNCTION, KEGG_TASTE_TRANSDUCTION, etc. (**Figure 4B**). Compared with subtype B, subtype C has more activated pathways, such as KEGG_GLYCOSAMINOGLYCAN_BIOSYNTHESIS_CHONDROITIN_SULFATE,KEGG_EPITHELIAL_CELL_SIGNALING_IN_HELICOBACTER_PYLORI_INFECTIO, etc. (**Supplementary Figure 1**)

### Risk-Score Model Based on ER-LncRNA Pairs

**Figure 5:**
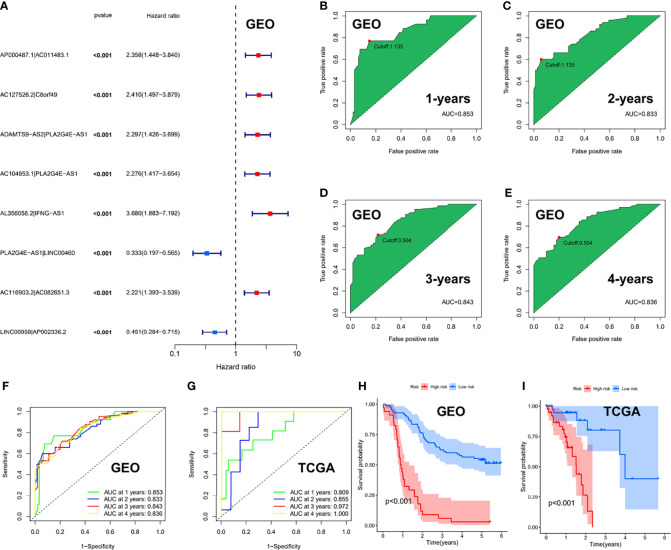
Risk-score model based on ER-lncRNA pairs in ESCC. **(A)** Forest plot of eight prognostic-related ER-lncRNA pairs through univariate Cox analysis in the GEO cohort. **(B-E)** The AUC value of the risk score. **(F, G)** ROC curve analysis of the accuracy of the model to predict patient prognosis at 1, 2, 3, and 4 years in the GEO cohort **(F)** and the TCGA cohort **(G)**. **(H, I)** Kaplan-Meier survival curves for ESCC patients from the GEO and TCGA cohorts, stratified according to cut-off value (high vs. low).

We performed univariate Cox regression analysis in the GEO cohort (*P* < 0.001) and identified 8 ER-lncRNA pairs with strong prognostic significance (**Figure 5A**). Eight ER-lncRNA pairs (AP000487.1|AC011483.1, AC127526.2|C8orf49, ADAMTS9-AS2|PLA2G4E-AS1, AC104653.1|PLA2G4E-AS1, AL356056.2|IFNG-AS1, PLA2G4E-AS1|LINC00460, AC116903.2|AC082651.3和LINC00958|AP002336.2) were included in the Cox regression model through LASSO regression analysis. Next, we calculated the AUC values of the risk-score model based on 8 pairs in the GEO cohort, where the cut-off values for 1 (**Figure 5B**), 2 (**Figure 5C**), 3 (**Figure 5D**), and 4 years (**Figure 5E**) were 1.135, 1.135, 0.504, and 0.504, respectively. Additionally, based on the same risk-score model, the validation set demonstrated superior survival prediction ability (**Figures 5F, G**). In addition, we grouped patients by their 1-year cut-off values in each cohort. Kaplan-Meier analysis showed that in the GEO cohort (**Figure 5H**) and the TCGA cohort (**Figure 5I**), patients in the low-risk group survived longer than those in the high-risk group (p < 0.001).

### Clinical Value and Application of Risk Score

**Figure 6:**
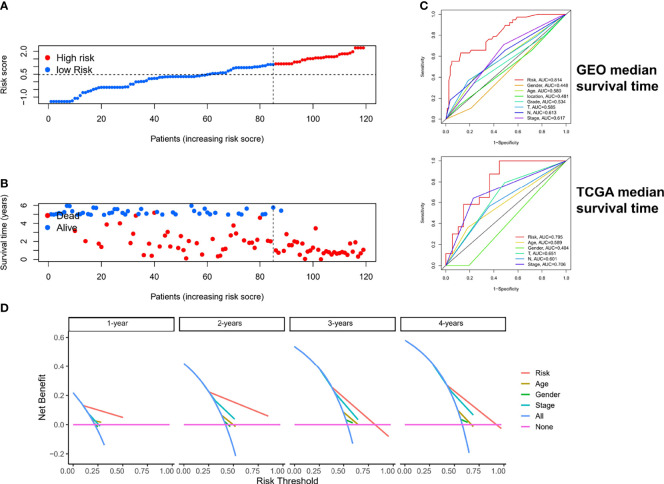
Estimation of clinical value of ER-lncRNA pairs risk-score model in ESCC patients. **(A)** Risk score distribution plot showed the distribution of high-risk and low-risk ESCC patients. **(B)** Scatter plot showed the correlation between the survival status and risk score of ESCC patients. **(C)** ROC curve analysis showed the prognostic accuracy of risk score and clinicopathological parameters. **(D)** Decision curve analysis (DCA).

The risk scores and survival rates for each case in the GEO cohort are shown in **Figures 6A, B**. These data suggest that patients in the low-risk group had better clinical outcomes than those in the high-risk group. In addition, ROC curve analysis showed that for median survival time, ER-lncRNA pairs–based risk stratification had better predictive indicative value than traditional clinical indicators (**Figure 6C**). The results of the DCA analysis also showed that ER-lncRNA pairs–based risk stratification had a better level of benefit relative to traditional clinical indicators (**Figure 6D**).

**Figure 7:**
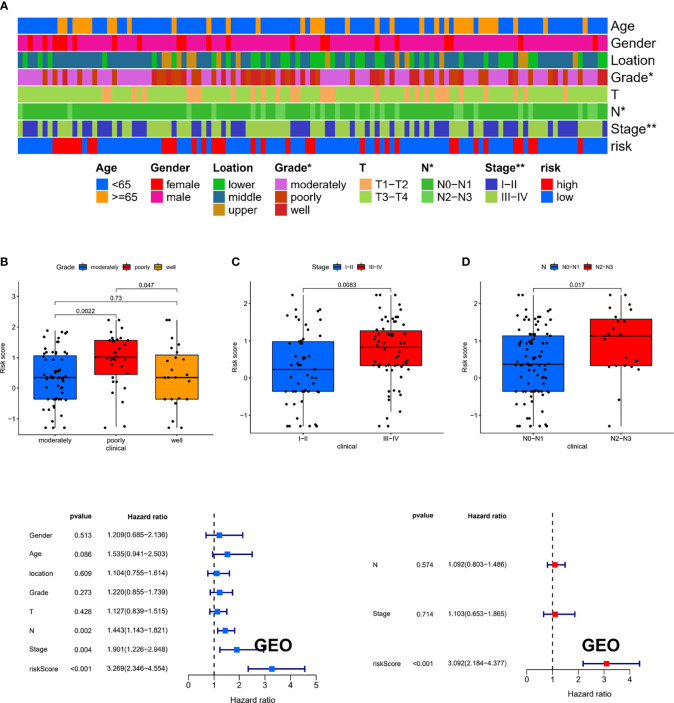
The clinical correlation between risk-score model and clinicopathological characteristics of ESCC patients. **(A)** The chi-square test showed that grade, N stage, and stage were significantly related to the risk-score model. The Wilcoxon rank-sum test showed that the risk scores of ESCC patients were significantly related to status of differentiation **(B)**, clinical stage **(C)**, and status of lymph node metastasis **(D)**. **(E, F)** The results of the univariate and multivariate Cox regression analyses regarding significant survival-related clinical characteristic parameters in GEO. *P < 0.05, **P < 0.01.

To estimate the correlation between risk-score model and clinicopathological characteristics of ESCC patients, we performed chi-square test in the GEO cohort, which showed that grade, N stage, and stage were significantly related to the risk-score model (**Figure 7A**). Furthermore, according to the Wilcoxon rank-sum test, the risk scores of ESCC patients were significantly related to clinical stage, status of differentiation, and status of lymph node metastasis (**Figures 7B–D**). Additionally, in the GEO cohort, we demonstrated that the risk score can be used as an independent prognostic factor for ESCC patients (**Figures 7E, F**). Consistent results were also exhibited in the TCGA cohort (**Supplementary Figure 2**).

### Risk-Score Model to Assess Tumor Microenvironment and Drug Sensitivity

**Figure 8:**
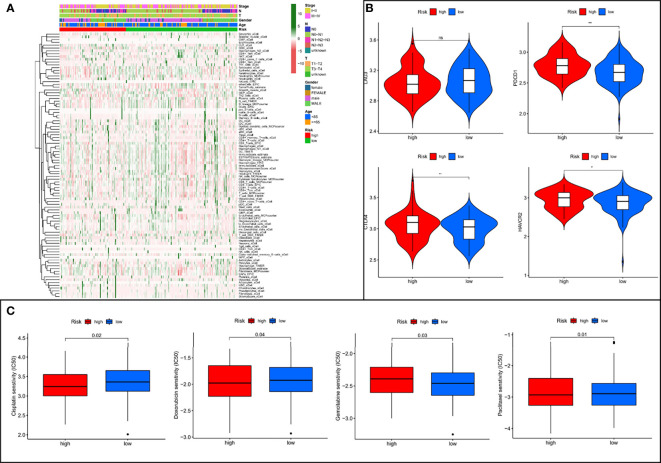
The low- and high-risk groups display different immune landscape, expression levels of immune checkpoint genes, and chemotherapy drug sensitivity. **(A)** Heat map showing the difference in the immune landscape between the low- and high-risk groups. **(B)** The expression levels of immune checkpoint genes between high- and low-risk groups. **(C)** The boxplot of sensitivity of common chemotherapy drugs between high- and low-risk groups. ns, not significant, *P < 0.05, **P < 0.01.

To further explore the relationship between risk assessment model and the tumor immune microenvironment, we analyzed the composition of immune cells in different risk subgroups in the meta cohort by applying multiple deconvolution algorithms. The results showed that the high-risk group had a higher landscape of immune cell infiltration (**Figure 8A**), as well as a more active TME (**Supplementary Figure 3**), compared to the low-risk group. In addition, in immune checkpoint mRNA expression, we found that high-risk patients had higher expression of PDCD1, CTLA4, and HAVCR2 (**Figure 8B**). Meanwhile, high-risk patients also had smaller IC50 values among most chemotherapeutic agents (**Figure 8C**), suggesting that the model could serve as a potential predictor of chemosensitivity.

### Risk Groupings Represent Different Microbiota Landscapes

**Figure 9:**
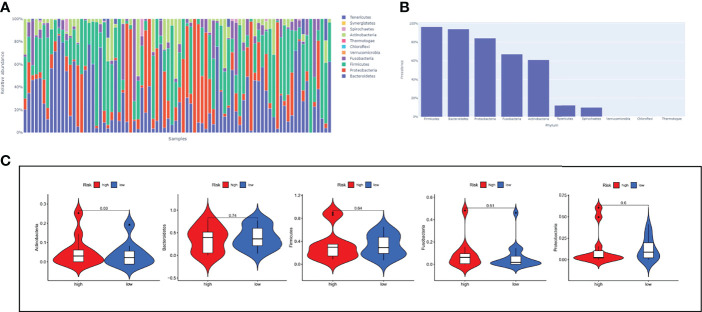
The relationship between risk-score model and microbiota landscapes. **(A)** Distribution of different sample flora. **(B)** The top five most abundant microbial phyla in ESCC. **(C)** The expression levels of five microbial phyla between high- and low-risk groups.

The Cancer Microbiome Atlas (TCMA) (23), which reveals the distribution of microbiota in TCGA sequenced samples, enables a systematically matched microbial-host multi-omics analysis. Our secondary analysis of the ESCC data among them founded that the ESCC environment contains the following five main microbial phyla: Firmicutes, Bacteroidetes, Proteobacteria, Fusobacteria, and Actinobacteria (**Figures 9A, B**). Interestingly, Actinobacteria was significantly upregulated in high-risk patients (**Figure 9C**), which may suggest us a large role of Actinobacteria in the development of ESCC.

### Identify the Best Biomarkers Based on Machine Learning Algorithms

**Figure 10:**
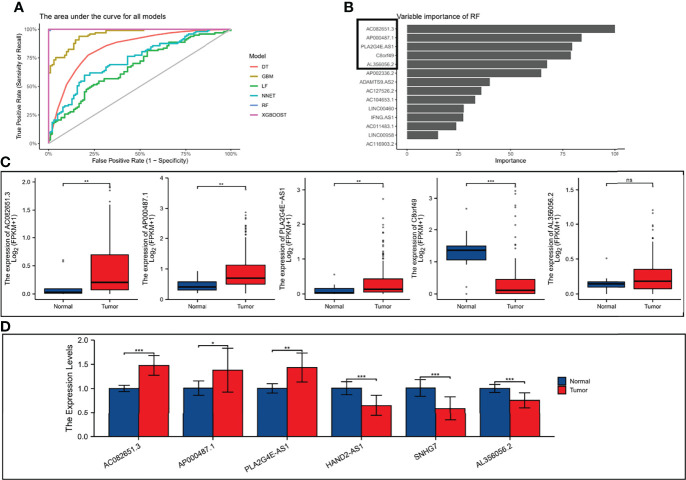
Validation the expression level of ER-lncRNAs. **(A)** The area under the curve for all models. **(B)** Top five importance of ER-lncRNAs in RF models. **(C)** The differential expression of AC082651.3, AP000487.1, PLA2G4E-AS1, C8orf49 and AL356056.2 in ESCC samples compared to adjacent normal controls in TCGA and GTEx RNA-seq data. **(D)** The expression levels of the 6 ER-lncRNAs in 10 pairs ESCC and matched adjacent normal tissues were examined by qRT-PCR. ns, not significant, *P < 0.05, **P < 0.01, ***P < 0.001.

We split the 8-pairs in the risk-score model and finally got 14 ER-lncRNAs. According to the method in the literature (24), with survival and death as binary dependent variables, based on the expression of 14 ER-lncRNAs, a variety of machine learning algorithms (DT, GBM, LF, NNET, RF and XGBOOST) were used for prediction. It was found that the AUCs of XGBOOST and RF were the largest (**Figure 10A**). In the RF model, the five most important ER-lncRNAs were: AC082651.3, AP000487.1, PLA2G4E-AS1, C8orf49 and AL356056.2 (**Figure 10B**). Subsequently, the expression levels of the above lncRNAs were analyzed by combining the GTEx and TCGA databases, in which only C8orf49 was upregulated in normal samples, and the rest of lncRNAs were upregulated in tumor samples (**Figure 10C**). In addition, qRT-PCR analysis based on clinical samples from our hospital showed a high degree of consistency (**Figure 10D**).

## Discussion

At present, a large number of research results have proved that the expression levels of many exosomal lncRNAs in pathological states, especially in neoplastic diseases, are significantly different from those in normal control samples. This indicates that exosomes can selectively package, secrete and transfer specific lncRNAs, and exert corresponding biological functions (19, 25). Exosomal lncRNA research is an important part of tumor biology, which can participate in the proliferation, invasion, metastasis, and angiogenesis of tumor cells, and also plays an important role in drug resistance and immunosuppressive microenvironment (26, 27). In addition, exosomal lncRNAs have been shown to regulate antigen presentation, affect immune cell activity, and induce apoptosis of relevant effector cells (17). Due to the above-mentioned properties of exosomal lncRNAs, they are now generally considered as novel biomarkers for early diagnosis, efficacy monitoring and prognostic assessment of tumors (20, 28). Therefore, a comprehensive study of prognostic significance of exosomal lncRNA in ESCC is essential.

To date, this is the first study to systematically elucidate the potential contribution of ER-lncRNAs in the prognosis of ESCC and specifically highlight their role in the tumor immune microenvironment. We screened 620 ER-lncRNAs using co-expression analysis, and then identified 142 differentially expressed ER-lncRNAs by secondary differential analysis. Next, we then determined two subtypes of ESCC—namely, cluster 1, cluster 2, and cluster 3—by consensus clustering based on the expression profiles of 3459 ER-lncRNA pairs from the meta cohort. The different subtypes significantly affect the prognosis and show significant differences in immunological characteristics, biological features and drug sensitivity. Subtype B was found to have the worst prognosis, while subtype A had the best prognosis. Subtype C had the highest stromal and immune score and the lowest tumor purity score, while subtype A had the lowest stromal and immune score and the highest tumor purity score. As predicted, subtype C had the highest enrichment and activity in immune cells, pathways or functions. Also, the mRNA levels of most of the immune checkpoints were higher in subtype C than in other subtypes. In contrast, subtype B is more sensitive to some classical chemotherapeutic agents. Herein, we speculated that exosome-related lncRNAs was associated with disease progression and immunotherapy in ESCC patients.

To improve the accuracy and validity of the prediction results, we developed a prognostic risk score model based on 8 ER-lncRNA pairs in the GEO cohort using univariate Cox regression analysis and LASSO Cox regression analysis. Next, the cut-off values were determined by evaluating the 1-year ROC curves in the training set (GEO cohort) and the validation set (TCGA cohort). Patients were divided into high-risk and low-risk groups according to the cut-off values. As expected, the results of survival analysis were consistent, and the low-risk group exhibited a better OS. Univariate and multivariate Cox regression analyses indicated that the risk score was an independent risk factor for survival in ESCC. Compared with other clinical biomarkers, such as TNM and stage, the risk score had better predictive indicative value. The clinicopathological characteristics, immune landscape, drug susceptibility, and microbiota landscapes were significantly different between the low- and high-risk groups through bioinformatic analysis. Finally, we split the 8-pairs in the risk-score model and finally got 14 ER-lncRNAs. According to the method in the literature (24), with survival and death as binary dependent variables, based on the expression of 14 ER-lncRNAs, a variety of machine learning algorithms (DT, GBM, LF, NNET, RF and XGBOOST) were used for prediction. In the RF model, the five most important ER-lncRNAs were: AC082651.3, AP000487.1, PLA2G4E-AS1, C8orf49 and AL356056.2. Subsequently, the expression levels of the above lncRNAs were analyzed by combining the GTEx and TCGA databases, in which only C8orf49 was upregulated in normal samples, and the rest of lncRNAs were upregulated in tumor samples. In addition, qRT-PCR analysis based on clinical samples from our hospital showed a high degree of consistency.

ESCC is regarded as an immunogenic tumor. Nevertheless, to a great extent, immune dysfunction is mediated by inducing immunosuppressive cells to infiltrate the TME (29, 30). For this purpose, we compared tumor-infiltrating immune cells among different subgroups, which serve as a powerful indicator to assess the tumor immune microenvironment. Similarly, immune infiltration cells were increased in the high-risk group compared with the low-risk group. The results showed that the high-risk group had a higher landscape of immune cell infiltration compared to the low-risk group.

Numerous clinical trials evaluating the role of immune checkpoint inhibitors (ICIs) in patients with ESCC are currently in progress. By examining the association between the risk score and expression of critical immune checkpoints, it was further observed that most immune checkpoints (4/4) exhibited higher expression in the high-risk group. Therefore, patients with high-risk scores might benefit more from ICIs compared to patients with low-risk scores. Chemotherapy remains the first-line treatment for advanced and metastatic ESCC. Owing to tumor heterogeneity in ESCC, the ESCC cases display variable sensitivity to chemotherapy. Therefore, we assessed the predictive value of the prognostic risk-score model for chemo-sensitivity in patients with ESCC. The IC50 results were encouraging:the high-risk group showed better efficacy for cisplatin, doxorubicin and paclitaxel than the low-risk group. These results suggest that risk score-based stratification may predict the efficacy of chemotherapy and immunotherapy and may help determine the most appropriate drug regimen for each ESCC patient.

Currently, the study of microbiota in life sciences has been greatly enhanced by advances in sequencing technology, accompanied by the application of multi-omics analysis (31). Meanwhile, a growing body of evidence suggests that alterations to the microbiome are associated with gastrointestinal cancer development, progression, and drug response (32, 33). Herein, we described the microbial signature associated with ESCC by conducting a comprehensive analysis of the bacterial taxa in the TCMA. Firmicutes, Bacteroidetes, Proteobacteria, Fusobacteria, and Actinobacteria dominated the top 5 abundant taxa at the phylum level, which is consistent with the results of other studies (34). In ESCC, the relative abundance of Firmicutes, Bacteroidetes, Proteobacteria and Fusobacteria in high-risk group was elevated (with no significant difference) when compared with low-risk group. The high-risk group had significantly higher levels of Actinobacteria compared to the low-risk group, which may suggest us a large role of Actinobacteria in the development of ESCC. We are sure that the use of lncRNA expression can reflect certain biological changes, but it does not fully reveal them, and this is where the limitation lies. In addition, it is well known that immune changes in ESCC are a dynamic process. However, due to limitation of data resource, we didn’t provide sample information and sampling time points for each patient.

This study fills the gap of ER-lncRNA model in predicting the prognosis of patients with ESCC and the risk score–based risk stratification could facilitate the determination of therapeutic options to improve prognoses.

## Data Availability Statement

The datasets presented in this study can be found in online repositories. The names of the repository/repositories and accession number(s) can be found in the article/**Supplementary Material**.

## Ethics Statement

The studies involving human participants were reviewed and approved by the Ethics Committee of The Second Hospital of Hebei Medical University. The patients/participants provided their written informed consent to participate in this study. Written informed consent was obtained from the individual(s) for the publication of any potentially identifiable images or data included in this article.

## Author Contributions

The detailed text is as follows: FZ, ZL, and SL conceived and designed the study. ZW, PG, and DZ collected and analyzed the data. FZ and ZD wrote the manuscript. All authors read and approved the manuscript.

## Funding

This study was supported by Science and Technology Plan Project of Hebei Province (No.223777106D) and General Project of Natural Science Foundation of Hebei Province (No. H2018206249).

## Conflict of Interest

The authors declare that the research was conducted in the absence of any commercial or financial relationships that could be construed as a potential conflict of interest.

## Publisher’s Note

All claims expressed in this article are solely those of the authors and do not necessarily represent those of their affiliated organizations, or those of the publisher, the editors and the reviewers. Any product that may be evaluated in this article, or claim that may be made by its manufacturer, is not guaranteed or endorsed by the publisher.

## Supplementary Material

Supplementary Figure 1 The GSVA pathway enrichment analysis between different subtypes.

Supplementary Figure 2 The results of the univariate and multivariate Cox regression analyses regarding significant survival-related clinical characteristic parameters in TCGA.

Supplementary Figure 3 Heat map showing the difference in the TME between the low- and high-risk groups.
