# Supplementary figures and images for: Exploring the Potential of Exosome-Related LncRNA Pairs as Predictors for Immune Microenvironment, Survival Outcome, and Microbiotain Landscape in Esophageal Squamous Cell Carcinoma
Source: Front Immunol. 2022 Jul 8;13:918154. doi: 10.3389/fimmu.2022.918154 (PMC9307964; doi:10.3389/fimmu.2022.918154)

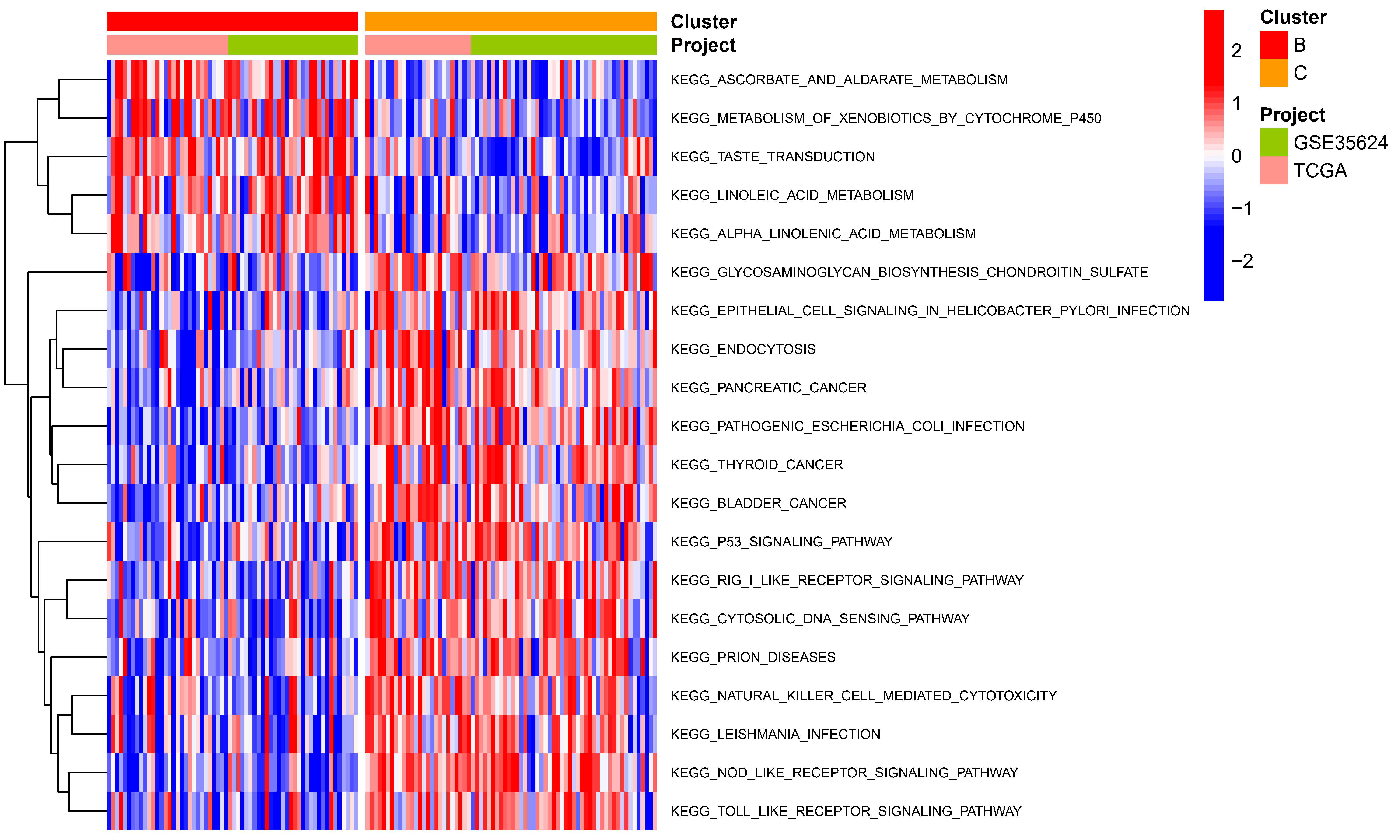

Supplement: Supplementary Figure 1 — The GSVA pathway enrichment analysis between different subtypes. [file Image_1.tiff]

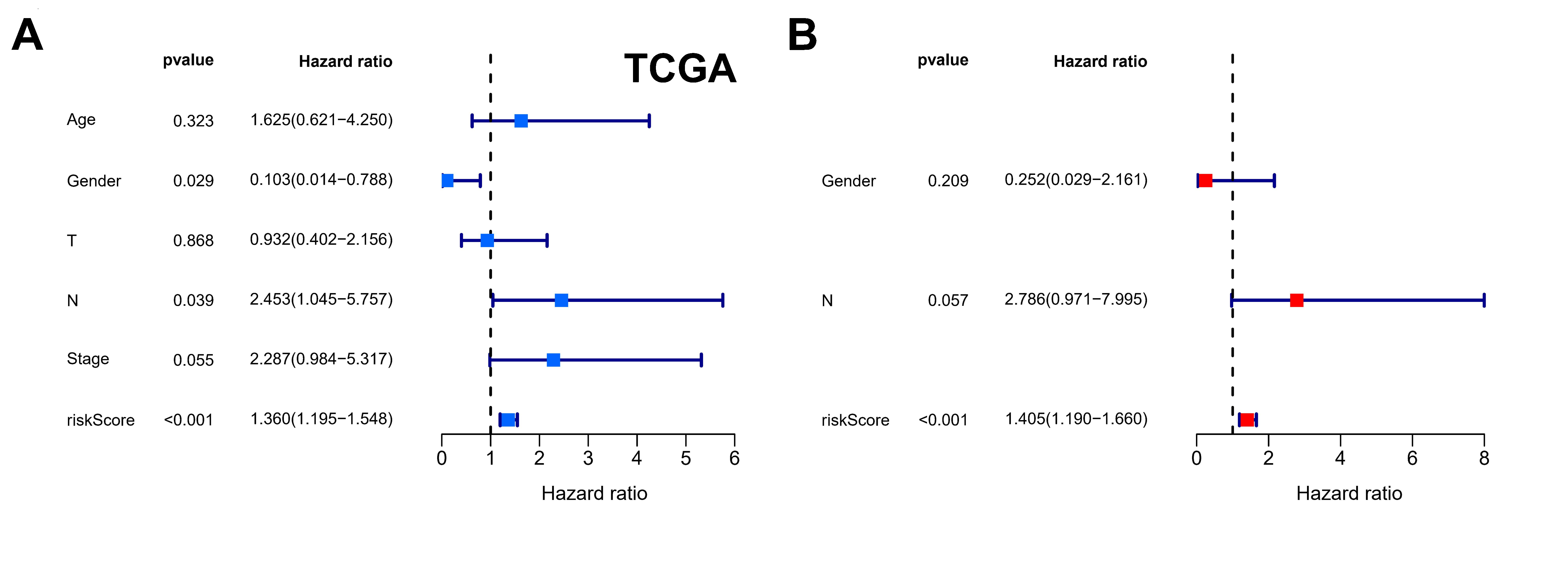

Supplement: Supplementary Figure 2 — The results of the univariate and multivariate Cox regression analyses regarding significant survival-related clinical characteristic parameters in TCGA. [file Image_2.tiff]

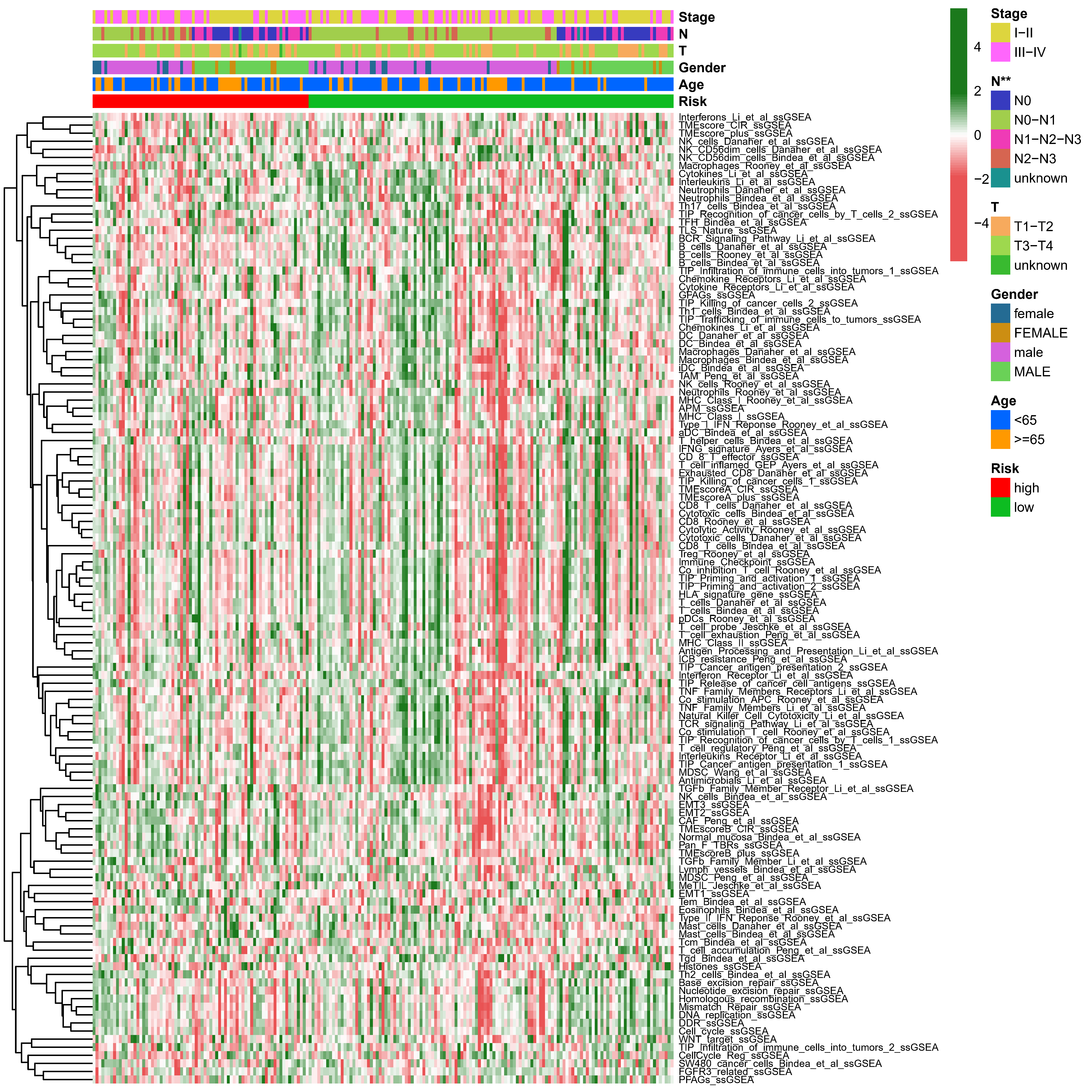

Supplement: Supplementary Figure 3 — Heat map showing the difference in the TME between the low- and high-risk groups. [file Image_3.tiff]
